# Association between intestinal worm infection and malnutrition among rural children aged 9–11 years old in Guizhou Province, China

**DOI:** 10.1186/s12889-019-7538-y

**Published:** 2019-09-02

**Authors:** Ming Guan, Bingxue Han

**Affiliations:** 1 Family Issues Center, Xuchang University, Road Bayi, Xuchang, 88 Henan China; 2 School of Business, Xuchang University, Road Bayi, Xuchang, 88 Henan China; 3 College of Urban and Rural Planning and Gardening, Xuchang University, Road Bayi, Xuchang, 88 Henan China

**Keywords:** Intestinal worm infection, Malnutrition, Low IQ, Anaemia, Socio-demographic factors

## Abstract

**Background:**

Intestinal worm infection adversely impacted child health and was one of the China’s largest health burdens. However, yet little was known about associations between intestinal worm infection and malnutrition in school-aged children in rural China. This study aimed to fill into the gap.

**Methods:**

Data were from a survey of children aged 9–11 years old in Guizhou Province, China conducted in June 2013. Considering anemia and low intelligent quotient (IQ) as mediating factors, binomial logistic regression was used to estimate the associations of intestinal worm infection with thinness, underweight, and stunting. Moreover, the associations between socio-demographic factors and malnutrition were also explored.

**Results:**

Among 2179 children, part of children was infected by intestinal worm (41.85%). Stunting (28%), low memory IQ (87.52%), and low process IQ (62.59%) were highly prevalent in the sample. Socio-demographic factors were associated with thinness, underweight, stunting, low memory IQ, low process IQ, anaemia, and intestinal worm infection. Intestinal worm infection was associated with low IQ, anemia, and stunting. In addition, anemia and low IQ could not confound the other expected associations.

**Conclusion:**

This study demonstrated the association between intestinal worm infections and stunting appeared to be largely mediated via low IQ. The study highlighted the importance of deworming and improving nutrition in the surveyed areas.

**Electronic supplementary material:**

The online version of this article (10.1186/s12889-019-7538-y) contains supplementary material, which is available to authorized users.

## Background

A growing number of studies showed intestinal worm infestation had negative associations with children’s health globally. In developing countries, worm infestation was an important cause of obscure acute gastrointestinal bleeding [1]. A study found worm infestation was negatively associated with eczema and allergic sensitization [2]. Furthermore, there was some evidence that acute idiopathic scrotal oedema in children was frequently associated with a history of intestinal worm infestation [3]. Specifically, roundworm infection could worsen eosinophilic pneumonia in children [4]. In rural Pakistan, malnutrition and anemia were highly prevalent and associated with parasitic infections in children [5]. Helminthiasis and giardiasis were associated with acute and chronic nutritional status respectively among the rural Venezuelan children [6]. Thus, it could speculate that intestinal worm infection could worsen the nutritional status of rural children.

Anthropometric indicators could assess the adequacy of diet and growth, in particular in children. For example, height-for-age could measure long-term improvements in child growth [7]. The European height-for-age charts were advocated to monitor growth of healthy and diseased European children [8]. In western countries, height for age measurement had potential in screening children for later risk of obesity [9]. A study in Zanzibari children reported hookworm-infected children had higher hemoglobin concentration (Hb) and mid-upper arm circumference-for-age Z score compared with uninfected children after adjusting for covariates [10]. Two of the most commonly used anthropometric indicators (eg., height-for-age and weight-for-age) could measure children’s nutritional status. In fact, rapid economic growth was not always followed by improved nutrition status of rural children in China [11]. According to definition of Shetty (2003), malnutrition covered undernutrition here [12]. To make matters worse, undernutrition in children under three and five years of age was confirmed [13, 14]. The causal effects had attracted attentions from the international academia. Here, the relationship between intestinal worm infestation and malnutrition was focused on. For this purpose, the mediating variables and control variables should be identified.

Socio-demographic factors were possibly control variables. The existing evidence suggested that socio-demographic factors were associated with anthropometric parameters. For instance, socio-economic gradients had an additional direct and independent effect on height, even in early childhood in the United States [15]. Similarly, the social milieu was important to understanding differences in height-for-age among children in Ghana [16]. Nutritional status and wealth could be modifiable factors to improve academic performance of school-aged children [17]. Also, specific socio-demographic characteristics were highly associated with infant anemia in rural areas of Shaanxi province, China [18]. Thus, when the relationship between intestinal worm infection and malnutrition was examined, socio-demographic factors could be viewed as control variables.

Anemia was a possible mediating variable. A study indicated that anemia had an important relation with anthropometric markers [19]. Another finding suggested that anemia was correlated with malnutrition in Croatian patients [20]. Partly due to undernutrition, individuals with sickle cell anemia showed slowed growth and delayed sexual maturity [21]. Thus, anemia could be considered as mediating variable of the relationship between intestinal worm infection and malnutrition.

Previous studies indirectly indicated that low IQ was also possibly a mediating variable. For instance, low IQ score in childhood was associated with obesity and weight gain in adulthood [22]. Low IQ was associated with increased odds of obesity in adolescents [23]. Several dietary studies indirectly revealed that there was relationship between nutrition and working memory. For example, dietary supplement use in old age had associations with childhood IQ, current cognition, and health [24]. A negative relation between body mass index (BMI) and working memory emerged in the high stereotype threat and the standard instructions conditions [25]. Another study suggested that better visuospatial working memory was associated with a preference for energy dense foods [26]. Previous studies indirectly reported that there was relationship between BMI and processing speed. For example, obese adolescents showed slower cognitive processing speed compared with their healthy weight peers [27]. There might be the detrimental influences of obesity on cognitive functioning in old age [28]. Thus, the speculation might be true.

The purpose of this study was to ascertain associations between intestinal worm infection and malnutrition after controlling for a number of confounders. With a publicly available data, the current study sought to fill identified epidemiological gaps in the knowledge regarding the prevalence of malnutrition expressed by thinness, underweight, and stunting among in-school children in China. Simultaneously, anemia and low IQ were depicted and assessed as mediators. Subsequently, the potential causal effects would be discovered in the joint with the relevant literature.

## Methods

### Data source

Data provided by Rozelle (2016) were from a survey of 9–11 years old students in Guizhou Province, China conducted in June 2013 [29]. Located in the southwestern part of China, Guizhou Province was a relatively poor, economically undeveloped, and mountainous province with most diverse ethnic minority groups. The survey covered 2246 children in 153 villages located across 7 counties in the province. The examination methods for worm were described by Liu, et al. (2015) [30].

### Main variables

Socio-demographic variables were taken as a dichotomous variables, including ethnic minority (1 = yes; 0 = no), gender (1 = male; 0 = female), grade (1 to 6), boarding (1 = yes; 0 = no), migrant mother (1 = yes; 0 = no), migrant father (1 = yes; 0 = no), household size, and number of siblings. Intestinal worm infection (1 = yes, 0 = no) denoted a student was infected by at least one of three types of intestinal worms (roundworm, hookworm, and whipworm).

Additional file 1: Table S1 showed development classification in children and adolescents aged 61 months to 19 years. Malnutrition was reflected by thinness measured by BMI-for-age z-score (BAZ), underweight measured by weight-for-age z-score (WAZ), and stunting measured by height-for-age z-score (HAZ). IQ was measured by Wechsler Intelligence Scale for Children with WISC-working memory index and WISC-process speed. Anaemia was measured by Hbs collected using HemoCue Hb 201+ fingerpick systems.

### Statistical analysis

The socioeconomic characteristics of the participants were estimated between infection status differences with chi-square test. Development characteristics of the sample populations were estimated between gender differences with chi-square test.

When analyzing the relationship between intestinal worm infection and malnutrition, low memory IQ, low process IQ, anemia (first), and anemia (altitude adjusted) could be considered as confounding variables. Here, socio-demographic variables like ethnic minority, gender, grade, boarding, migrant mother, and migrant father were considered as control variables. Due to the statistical similarities between mediation and confounding [31], Stata program *medeff (logit)* was adopted to explore how intestinal worm infection influenced malnutrition. The statistical analysis was conducted in three scenarios on the basis of malnutrition categories.

All analyses were performed in Stata/MP 14.0 (StataCorp, College Station, TX).

## Results

In Table 1, the percentage of male infected by worms was higher than that of female infected by worms. Most of them were children with ethnic minority. Nearly 40% of children were left behind by their parents in the villages. About a quarter of them were boarded at school. The intestinal worm infection was more prevalent in males than that in females. There were significant differences between uninfected and infected children with respect to grade, boarding, migrant mother, and migrant father. Thus, it was speculated a vast majority of sampled children lived in the remote villages because rural residents with ethnic minority often distributed in the poverty-stricken mountain villages. Also, part of them was poor since their parents migrated out of the villages into cities for jobs due to income gaps between rural and urban places.

**Table 1:** Sample characteristics among respondents uninfected and infected by intestinal worms, n (%)

Characteristics	Uninfected (%)	Infected (%)	Chi-square	*P* value
Gender (*N* = 2179)			2.2062	0.137
Female	602 (27.63%)	404 (18.54%)		
Male	665 (30.52%)	508 (23.31%)		
Ethnic minority (N = 2179)			0.0988	0.753
No	126 (5.78%)	87 (3.99%)		
Yes	1141 (52.36%)	825 (37.86%)		
Grade (N = 2179)			4.4469	0.035**
1–3	559 (25.65%)	444 (20.38%)		
4+	708 (32.49%)	468 (21.48%)		
Boarding (*N* = 2175)			9.6265	0.002***
No	966 (44.41%)	641 (29.47%)		
Yes	299 (13.75%)	269 (12.37%)		
Migrant mother (N = 2179)			22.0939	0.000***
No	717 (32.91%)	607 (27.86%)		
Yes	550 (25.24%)	305 (14.00%)		
Migrant father (N = 2179)			20.4335	0.000***
No	683 (31.34%)	580 (26.62%)		
Yes	584 (26.80%)	332 (15.24%)		
Household size (N = 2179)			2.4913	0.114
2–4	382 (17.53%)	304 (13.95%)		
5+	885 (40.61%)	608 (27.90%)		
Number of siblings (N = 2179)			0.0590	0.808
0	282 (12.94%)	207 (9.50%)		
1+	985 (45.20%)	705 (32.35%)		

Note: *** and ** indicated 0.01 and 0.05 significance level, respectively

Development characteristics of the children were shown in Additional file 2: Table S2. These school-aged children were at risk of thinness (6.56%), underweight (5.83%), stunting (27.99%), low memory IQ (87.52%), low process IQ (62.60%), anaemia (first) (18.08%), anaemia (altitude adjusted) (16.84%), and intestinal worm infection (41.85%). There were no significant differences between genders with respect to thinness, underweight, stunting, low memory IQ, anaemia (first), and anaemia (altitude adjusted). But, there was significant difference between genders with regard to low process IQ. The means of BAZ, WAZ, HAZ, and Hb were higher than their cut-off values. But, the means of WISC - working memory and WISC - process speed were lower than their cut-off values.

See Table 2. In models 2, 3, and 4, after controlling for the socio-demographic variables, intestinal worm infection was significantly associated with anemia (altitude adjusted), low process IQ, and low memory IQ. Among the socio-demographic variables, ethnic minority was a significant predictor of thinness in models 1, 2, 3, and 4. Gender was a significant predictor of thinness in model 3. Grade was a significant predictor of anemia (first), anemia (altitude adjusted), low process IQ, and low memory IQ in models 1, 2, 3, and 4. Boarding was a significant predictor of low memory IQ in model 4. Migrant mother and migrant father were significant predictors of low process IQ in model 3. Household size was significantly associated with anemia (first), anemia (altitude adjusted), and low memory IQ in models 1, 2, and 4.

**Table 2:** Associations between intestinal worm infection and thinness confounded by anemia and low IQ

	Model 1		Model 2		Model 3		Model 4	
	Anaemia (first)	Thinness	Anaemia (altitude adjusted)	Thinness	Low process IQ	Thinness	Low memory IQ	Thinness
Intestinal worm infection	0.19 (0.11)	0.22 (0.18)	0.24** (0.12)	0.22 (0.18)	0.61*** (0.09)	0.22 (0.18)	0.81*** (0.15)	0.22 (0.18)
Low memory IQ								0.01 (0.27)
Low process IQ						0.01 (0.18)		
Anaemia (altitude adjusted)				0.17 (0.22)				
Anaemia (first)		0.18 (0.22)						
Ethnic minority	−0.11 (0.18)	1.16** (0.46)	− 0.11 (0.19)	1.16** (0.46)	− 0.11 (0.15)	1.15** (0.46)	− 0.05 (0.23)	1.15** (0.46)
Gender	−0.08 (0.11)	0.13 (0.18)	−0.06 (0.12)	0.13 (0.18)	0.16* (0.09)	0.13 (0.18)	0.08 (0.13)	0.13 (0.18)
Grade	−0.16*** (0.06)	0.03 (0.09)	−0.18*** (0.06)	0.03 (0.09)	−0.25*** (0.05)	0.02 (0.09)	−0.11* (0.07)	0.02 (0.09)
Boarding	0.08 (0.13)	−0.18 (0.21)	0.05 (0.13)	−0.18 (0.21)	0.05 (0.10)	−0.18 (0.21)	0.50*** (0.17)	−0.18 (0.21)
Migrant mother	0.07 (0.14)	0.14 (0.22)	0.11 (0.14)	0.14 (0.22)	−0.19* (0.11)	0.14 (0.22)	−0.01 (0.16)	0.14 (0.22)
Migrant father	−0.03 (0.14)	0.05 (0.21)	−0.06 (0.14)	0.05 (0.21)	0.22* (0.11)	0.05 (0.21)	−0.05 (0.16)	0.05 (0.21)
Household size	−0.12** (0.06)	−0.12 (0.09)	− 0.15** (0.06)	−0.12 (0.09)	− 0.06 (0.04)	−0.12 (0.09)	− 0.11* (0.06)	−0.12 (0.09)
Number of siblings	−0.08 (0.08)	0.08 (0.13)	−0.03 (0.09)	0.08 (0.13)	−0.03 (0.06)	0.08 (0.13)	0.01 (0.09)	0.08 (0.13)
Constant	−0.19 (0.37)	−3.54*** (0.69)	−0.15 (0.38)	− 3.54*** (0.69)	1.49*** (0.30)	− 3.48*** (0.70)	2.61*** (0.43)	− 3.48*** (0.73)
Pseudo R^2^	0.0135	0.0143	0.0156	0.0142	0.0326	0.0137	0.0355	0.0137
Observations	2175	2175	2175	2175	2175	2175	2175	2175

Note: ***, ** and * indicates 0.01, 0.05, and 0.10 significance level, respectively

See Table 3. In models 1, 2, 3, and 4, after controlling for the socio-demographic variables, intestinal worm infection was significantly associated with anemia (altitude adjusted), low process IQ, and low memory IQ. Among the socio-demographic variables, gender was a significant predictor of underweight in model 3. Grade was a significant predictor of anemia (first), anemia (altitude adjusted), low process IQ, and low memory IQ in models 1, 2, 3, and 4. Grade was significantly associated with underweight in models 1, 2, 3, and 4. Boarding was a significant predictor of low memory IQ in model 4. Migrant mother and migrant father were significant predictors of low process IQ in model 3, respectively. Household size was significantly associated with anemia (first), anemia (altitude adjusted), and low memory IQ in models 1, 2, and 4. Similarly, household size was significantly associated with underweight in models 1, 2, 3, and 4.

**Table 3:** Associations between intestinal worm infection and underweight confounded by anemia and low IQ

	Model 1		Model 2		Model 3		Model 4	
	Anaemia (first)	Underweight	Anaemia (altitude adjusted)	Underweight	Low process IQ	Underweight	Low memory IQ	Underweight
Intestinal worm infection	0.19 (0.11)	0.18 (0.20)	0.24** (0.12)	0.18 (0.20)	0.61*** (0.09)	0.20 (0.20)	0.81*** (0.15)	0.20 (0.20)
Low memory IQ								−0.17 (0.30)
Low process IQ						−0.13 0.21)		
Anaemia (altitude adjusted)				0.14 (0.25)				
Anaemia (first)		0.17 (0.24)						
Ethnic minority	−0.11 (0.18)	0.60 (0.42)	−0.11 (0.19)	0.60 (0.42)	−0.11 (0.15)	0.59 (0.42)	−0.05 (0.23)	0.59 (0.42)
Gender	−0.08 (0.11)	0.10 (0.20)	−0.06 (0.12)	0.10 (0.20)	0.16* (0.09)	0.10 (0.20)	0.08 (0.13)	0.10 (0.20)
Grade	−0.16*** (0.06)	−1.47*** (0.13)	−0.18*** (0.06)	−1.46*** (0.13)	− 0.25*** (0.05)	− 1.47*** (0.13)	−0.11* (0.07)	−1.46*** (0.13)
Boarding	0.08 (0.13)	−0.19 (0.23)	0.05 (0.13)	−0.19 (0.23)	0.05 (0.10)	−0.19 (0.23)	0.50*** (0.17)	−0.18 (0.23)
Migrant mother	0.07 (0.14)	−0.16 (0.25)	0.11 (0.14)	−0.16 (0.25)	−0.19* (0.11)	− 0.16 (0.25)	−0.01 (0.16)	− 0.16 (0.25)
Migrant father	−0.03 (0.14)	− 0.10 (0.24)	−0.06 (0.14)	− 0.10 (0.24)	0.22* (0.11)	− 0.10 (0.24)	−0.05 (0.16)	− 0.10 (0.24)
Household size	−0.12** (0.06)	0.17* (0.09)	−0.15** (0.06)	0.17* (0.09)	−0.06 (0.04)	0.17* (0.09)	−0.11* (0.06)	0.17* (0.09)
Number of siblings	−0.08 (0.08)	−0.10 (0.13)	− 0.03 (0.09)	−0.10 (0.13)	− 0.03 (0.06)	−0.10 (0.13)	0.01 (0.09)	−0.10 (0.13)
Constant	−0.19 (0.37)	0.40 (0.67)	−0.15 (0.38)	0.42 (0.67)	1.49*** (0.30)	0.56 (0.69)	2.61*** (0.43)	0.61 (0.72)
Pseudo R^2^	0.0135	0.1908	0.0156	0.1906	0.0326	0.1906	0.0355	0.1906
Observations	2175	2175	2175	2175	2175	2175	2175	2175

Note: ***, ** and * indicated 0.01, 0.05, and 0.10 significance level, respectively

See Table 4. In models 2, 3, and 4, after controlling for the socio-demographic variables, intestinal worm infection was significantly associated with anemia (altitude adjusted), low process IQ, and low memory IQ. Intestinal worm infection was significantly associated with stunting in models 1, 2, 3, and 4. Low memory IQ and low process IQ were significantly associated with stunting in models 3 and 4. Thus, low memory IQ and low process IQ confounded the relationships between intestinal worm infection and stunting, respectively.

**Table 4:** Associations between intestinal worm infection and stunting confounded by anemia and low IQ

	Model 1		Model 2		Model 3		Model 4	
	Anaemia (first)	Stunting	Anaemia (altitude adjusted)	Stunting	Low process IQ	Stunting	Low memory IQ	Stunting
Intestinal worm infection	0.19 (0.11)	0.49*** (0.10)	0.24** (0.12)	0.49*** (0.10)	0.61*** (0.09)	0.42*** (0.10)	0.81*** (0.15)	0.44*** (0.10)
Low memory IQ								0.75*** (0.18)
Low process IQ						0.59*** (0.11)		
Anaemia (altitude adjusted)				0.16 (0.13)				
Anaemia (first)		0.10 (0.12)						
Ethnic minority	−0.11 (0.18)	0.24 (0.17)	−0.11 (0.19)	0.24 (0.17)	−0.11 (0.15)	0.24 (0.17)	−0.05 (0.23)	0.24 (0.17)
Gender	−0.08 (0.11)	−0.16 (0.10)	− 0.06 (0.12)	−0.16 (0.10)	0.16* (0.09)	−0.18* (0.10)	0.08 (0.13)	−0.17* (0.10)
Grade	−0.16*** (0.06)	−0.19*** (0.05)	− 0.18*** (0.06)	−0.19*** (0.05)	− 0.25*** (0.05)	−0.16*** (0.05)	− 0.11* (0.07)	−0.19*** (0.05)
Boarding	0.08 (0.13)	0.32*** (0.11)	0.05 (0.13)	0.32*** (0.11)	0.05 (0.10)	0.32*** (0.11)	0.50*** (0.17)	0.29*** (0.11)
Migrant mother	0.07 (0.14)	0.01 (0.12)	0.11 (0.14)	0.01 (0.12)	−0.19* (0.11)	0.03 (0.12)	−0.01 (0.16)	0.01 (0.12)
Migrant father	−0.03 (0.14)	0.03 (0.12)	−0.06 (0.14)	0.03 (0.12)	0.22* (0.11)	0.00 (0.12)	−0.05 (0.16)	0.03 (0.12)
Household size	−0.12** (0.06)	−0.05 (0.05)	− 0.15** (0.06)	−0.05 (0.05)	− 0.06 (0.04)	−0.04 (0.05)	− 0.11* (0.06)	−0.04 (0.05)
Number of siblings	−0.08 (0.08)	0.14** (0.07)	−0.03 (0.09)	0.14** (0.07)	−0.03 (0.06)	0.15** (0.07	0.01 (0.09)	0.14** (0.07)
Constant	−0.19 (0.37)	−0.65** (0.33)	− 0.15 (0.38)	−0.67** (0.33)	1.49*** (0.30)	−1.11 *** (0.34)	2.61*** (0.43)	−1.31*** (0.37)
Pseudo R^2^	0.0135	0.0254	0.0156	0.0258	0.0326	0.0372	0.0355	0.0328
Observations	2175	2175	2175	2175	2175	2175	2175	2175

Note: ***, ** and * indicates 0.01, 0.05, and 0.10 significance level, respectively

Among the socio-demographic variables, gender was a significant predictor of low process IQ and stunting in model 3 and stunting in model 4. Grade was a significant predictor of anemia (first), anemia (altitude adjusted), low process IQ, and low memory IQ in models 1, 2, and 3. Grade was significantly associated with stunting in models 1, 2, 3, and 4. Boarding was a significant predictor of low memory IQ in model 4 and stunting in models 1, 2, 3, and 4. Migrant mother and migrant father were significant predictors of low process IQ in model 3. Household size was significantly associated with anemia (first), anemia (altitude adjusted), and low memory IQ in models 1, 2, and 4. Number of siblings was a significant predictor of stunting in models 1, 2, 3, and 4.

## Discussion

In all, this study analyzed the association between intestinal worm infection and malnutrition in Guizhou Province, China. The children at 9–11 years of age were highly at risk of malnutrition, low IQ, and anaemia. There was also high prevalence of intestinal worm infection among them. Socio-demographic variables had significant associations with malnutrition, low IQ, and anemia. Intestinal worm infection had significant associations with malnutrition, low IQ, and anemia. Anemia had no significant associations with low IQ and malnutrition. Moreover, low IQ could confound the associations between intestinal worm infection and stunting. Associations between intestinal worm infection and thinness and associations between intestinal worm infection and underweight could not be confounded by anemia and low IQ. Associations between intestinal worm infection and stunting could not be confounded by anemia.

This study was in line with an early study that the burden of children malnutrition could be aggravated by infestation with hookworms [32]. A review reported intestinal worm infection could be due to antihygienic lifestyle [33]. Another study indicated that the high prevalence of intestinal parasitic infections and anemia could translate as indiscriminate defecation, low socio-economic status, ignorance, and low standard of personal hygiene [34]. Although local governments made great efforts to improve child development, they did not promote equity of nutrition [35]. From 2006 to 2009, comprehensive control in demonstration plots of parasitic diseases in Guizhou Province took limited effects [36]. The other causes might be lack of clean water and sewage treatment facility.

With respect to anemia, the results from this study were in line with the prior studies. For example, the presence of worms in marginally nourished children could contribute significantly to blood loss in the intestine with resultant anemia [37]. There was a significant correlation between anemia and worm infestation [38]. Clinically, weekly iron-folic acid along with deworming had beneficial effect on the hemoglobin levels of the children [39].

Regarding socio-demographic factors, the findings from this study were in agreement with the prior studies. For example, socio-demographic factors and possibly parasitic infections intertwined to cause these anemia and malnutrition in the Peruvian highlands [40]. A cross-sectional assessment in children and adolescents aged 0–17 years concluded socioeconomic-related factors might contribute to the existence of stunting [41]. This study also accorded with the result in north Gaza Strip that there were socio-demographic differences in nutritional status among school adolescents aged 12 to 15 years [42]. In congruence with a study in northern Ethiopia, socioeconomic factors were significantly associated with children undernutrition [43]. Also, poor socioeconomic status had an adverse effect on the nutritional status and hemoglobin of sickle cell anemia patients [44].

Notably, socio-demographic factors interpreted the health care inequity among the children. First of all, boarding had significant association with stunting and low memory IQ. This was because food and living conditions provided by rural boarding schools were worse than those from parental home. Even more worse, unsafe food and abusive events often happened to boarding children in rural schools. Thus, parental care might be the best care harbor for their children. Secondly, ethnic minority had significant associations with thinness rather than underweight and stunting. This could be explained by the fact that ethnic children could not gain balanced diet in the poor, underserved, remote, and border areas. Finally, data analysis showed that grade negatively predicted low IQ, anemia, underweight, and stunting. This indicated children with high grade might have higher health literacy and better health status compared with students with low grade.

On the basis of the early studies, the present study enriched the knowledge of the relationship between anemia and malnutrition. An understanding of the risk factors for anemia and malnutrition among a population was fundamental to provide efficient preventive and control measures [45]. Anemia might be due to malnutrition rather than to diseases [46]. Prior studies reported that malnutrition had significant associations with anemia. The indirect relationships also were reported among Brazilian school children [47], from an Mexican incentive-based development program [48], and in Cambodian infants [49]. In India, severe anemia was one of the comorbidities responsible for severe malnutrition among children [50]. A cross-sectional study in rural Chadian children also reported that malnutrition was significantly associated with selected intestinal parasites and anemia [51].

Here, low IQ might influence malnutrition via mental disorders. Literature [52] demonstrated the importance of cultivating intelligence of children with ethnic minority. Low IQ was one of the risk factors for the children, which could be confirmed by the results from England and Iraq. A study in England showed those persons with lower IQ were less happy than those with higher IQ [53]. Another study in Iraq reported child IQ was found to be associated with the mental stress and service domains of the living environment [54]. Thus, the children left behind by their fathers or mothers might be involved in mental distress. Anorexia and other eating disorders driven by psychological disorders might be prevalent among the children.

On the basis of the early studies, the current study indicated that there might exist bidirectional associations between stunting and low IQ. More specifically, malnutrition at an early age might affect brain development [55] and was associated with poor long-term cognitive development in school-age children [56]. Prior studies also suggested that cognitive development in children benefited from specific preventive nutritional interventions [57], such as regular breakfast consumption [58]. This study was in line with the prior studies with respect to the relationship between stunting and child development [59–61]. More importantly, stunting in early childhood was associated with cognitive and educational deficits in late adolescence [62]. Likewise, a previous study reported the association between intelligence and diet might be beneficial to children’s cognitive development [63]. Thus, the triangle relationships among malnutrition, low IQ, and dietary could be possible.

On the shoulder of the previous studies, this study deepened the understanding of the relationship between anemia and IQ development. Several prior studies confirmed children’s cognitive development depended on Hb. For example, a Chinese study discovered high maternal Hb during early gestation might adversely affect children’s cognitive development [64]. Experimentally, hemoglobin level was linked to growth hormone-dependent proteins in short children [65]. Empirically, this study did not show the direct relationship between them.

The findings from this study were in line with those in other provinces in China. For example, a cross-sectional study conducted in the poor rural areas in Guangxi Autonomous Regional and Hainan Province showed that soil transmitted helminths (STHs) infection was one of the important risk factors for stunting [66]. Another study conducted in 141 impoverished rural areas of Guizhou and Sichuan Provinces showed that STH infection was associated with significantly lower WAZ and HAZ [67]. The significantly impaired physical fitness of school-aged children who were stunted or infected with STHs was confirmed in rural southwest China [68]. Since STH reinfections [69, 70] often occurred after treatment in the neighboring provinces, Guizhou province could learn the controlling experiences from Yunnan province [71] and Jiangxi province [72].

There were two limitations in this study. First, this study was limited to measuring wasting of children. This was because Rozelle (2016) did not expound the computing process of anthropometric references of weight-for-age, height-for-age, and bmi-for-age [29]. Second, the mediating relationship need further be explored, since statistical program with multiple mediators has not been developed till now.

## Conclusion

The present study showed an overall alarming situation of stunting, low IQ, and intestinal worm infection among children aged 9–11 years old in Guizhou Province. Statistically, low IQ had mediating effects on the associations between intestinal worm infection and stunting. The current study highlighted the importance of improving socioeconomic status, need for deworming, and improving the physical conditions among the in-school children. The findings from this study would help local authorities in their efforts to deworm, alleviate poverty, develop sanitation, and improve nutritional status of children.

## Additional files

Additional file 1: **Table S1.** Development classification in children and adolescents aged 61 months to 19 years. Note: ^a^ WHO standards [73];^b^ Wechsler Intelligence Scale for Children–Fifth Edition (WISC-V) IQ classification [74]; ^c^ WHO’s hemoglobin thresholds used to define anemia [75]. (DOC 36 kb)

Additional file 2: **Table S2.** Development characteristics of the sample populations. Note: ** indicated 0.05 significance level. (DOC 61 kb)

## Data Availability

Access to the survey data is open and publicly available in the following link. https://dataverse.harvard.edu/dataset.xhtml?persistentId=doi:10.7910/DVN/V3DVQ9.

## Abbreviations

BAZ: BMI-for-age z-score
HAZ: Height-for-age z-score
Hb: Hemoglobin concentration
IQ: Intelligent quotient
WAZ: Weight-for-age z-score
