# Supplementary material for: Association between intestinal worm infection and malnutrition among rural children aged 9–11 years old in Guizhou Province, China
Source: BMC Public Health. 2019 Sep 2;19:1204. doi: 10.1186/s12889-019-7538-y (PMC6719348; doi:10.1186/s12889-019-7538-y)
Supplement: Supplementary file 1 — Table S1. Development classification in children and adolescents aged 61 months to 19 years. Note: a WHO standards [73];b Wechsler Intelligence Scale for Children–Fifth Edition (WISC-V) IQ classification [74]; c WHO’s hemoglobin thresholds used to define anemia [75]. (DOC 36 kb) [file 12889_2019_7538_MOESM1_ESM.doc]

Table S1. Development classification in children and adolescents aged 61 months to 19 years.

| Indicators | cut-off values |
| --- | --- |
| Thinness^a^ | BAZ < –2 SD |
| Underweight^a^ | WAZ <–2SD |
| Stunting^a^ | HAZ <–2SD |
| Low memory IQ^b^ | IQ<90 |
| Low process IQ^b^ | IQ<90 |
| Anaemia (first) ^c^ | Hb<=115 g/d |
| Anaemia (altitude adjusted)^c^ | Hb<=115 g/d |

Note: ^a^ WHO standards ^31^;^b^ Wechsler Intelligence Scale for Children–Fifth Edition (WISC-V) IQ classification ^32^; ^c^ WHO's hemoglobin thresholds used to define anemia ^33^.
