# Supplementary material for: Association between intestinal worm infection and malnutrition among rural children aged 9–11 years old in Guizhou Province, China
Source: BMC Public Health. 2019 Sep 2;19:1204. doi: 10.1186/s12889-019-7538-y (PMC6719348; doi:10.1186/s12889-019-7538-y)
Supplement: Supplementary file 2 — Table S2. Development characteristics of the sample populations. Note: ** indicated 0.05 significance level. (DOC 61 kb) [file 12889_2019_7538_MOESM2_ESM.doc]

Table S2. Development characteristics of the sample population.

|  | Total | Female (%) | Male (%) | Chi-square | P value | Mean | Std. Dev. | Min | Max |
| --- | --- | --- | --- | --- | --- | --- | --- | --- | --- |
| BAZ | 2177 |  |  |  |  | -0.591 | 0.992 | -5.04 | 3.24 |
| Thinness | 2179 |  |  | 0.7589 | 0.384 |  |  |  |  |
| No |  | 43.37 | 50.07 |  |  |  |  |  |  |
| Yes |  | 2.80 | 3.76 |  |  |  |  |  |  |
| WAZ | 680 |  |  |  |  | -1.127 | 1.026 | -3.63 | 2.42 |
| Underweight | 2179 |  |  | 0.0898 | 0.764 |  |  |  |  |
| No |  | 43.55 | 50.62 |  |  |  |  |  |  |
| Yes |  | 2.62 | 3.21 |  |  |  |  |  |  |
| HAZ | 2178 |  |  |  |  | -1.390 | 1.044 | -8.37 | 2.85 |
| Stunting | 2179 |  |  | 2.4564 | 0.117 |  |  |  |  |
| No |  | 32.49 | 39.51 |  |  |  |  |  |  |
| Yes |  | 13.68 | 14.32 |  |  |  |  |  |  |
| WISC - working memory | 2179 |  |  |  |  | 78.595 | 9.932 | 45 | 147 |
| Low memory IQ | 2179 |  |  |  |  |  |  |  |  |
| No |  | 6.15 | 6.33 |  |  |  |  |  |  |
| Yes |  | 40.02 | 47.50 |  |  |  |  |  |  |
| WISC - process speed | 2179 |  |  |  |  | 86.155 | 13.108 | 45 | 138 |
| Low process IQ | 2179 |  |  | 5.6356 | 0.018** |  |  |  |  |
| No |  | 18.49 | 18.91 |  |  |  |  |  |  |
| Yes |  | 27.67 | 34.92 |  |  |  |  |  |  |
| Hb | 2179 |  |  |  |  | 126.251 | 12.446 | 72 | 157 |
| Anemia (first) | 2179 |  |  | 0.0001 | 0.991 |  |  |  |  |
| No |  | 37.82 | 44.10 |  |  |  |  |  |  |
| Yes |  | 8.35 | 9.73 |  |  |  |  |  |  |
| Anemia (altitude adjusted) | 2179 |  |  | 0.0272 | 0.869 | 126.239 | 12.435 | 72.079 | 156.659 |
| No |  | 38.46 | 44.70 |  |  |  |  |  |  |
| Yes |  | 7.71 | 9.13 |  |  |  |  |  |  |
| Intestinal worm infection | 2179 |  |  | 2.2062 | 0.137 | 0.419 | 0.493 | 0 | 1 |
| No |  | 27.63 | 30.52 |  |  |  |  |  |  |
| Yes |  | 18.54 | 23.31 |  |  |  |  |  |  |

Note: ** indicated 0.05 significance level.
